# Associations of triglyceride-glucose index and metabolic score for insulin resistance with various hypertension phenotypes in children and adolescents: results from the 2017 China nutrition and health surveillance

**DOI:** 10.3389/fendo.2025.1595097

**Published:** 2025-07-24

**Authors:** Haiyuan Zhu, Lianlong Yu, Qiqi Wu, Runquan Zhang, Zebang Zhang, Yumei Feng, Tao Liu, Dan Liu, Jiewen Peng, Xiongfei Chen, Xiaomei Dong

**Affiliations:** ^1^ Department of Public Health and Preventive Medicine, School of Medicine, Jinan University, Guangzhou, China; ^2^ Health Management Institute, Shandong Center for Disease Control and Prevention, Jinan, China; ^3^ Department of Epidemiology, School of Public Health, Southern Medical University, Guangzhou, China; ^4^ Guangdong Provincial Institute of Public Health, Guangdong Center for Disease Control and Prevention, Guangzhou, China; ^5^ Department of Primary Public Health, Guangzhou Center for Disease Control and Prevention, Guangzhou, China

**Keywords:** adolescents, children, insulin resistance, TyG, METS-IR, hypertension, blood pressure

## Abstract

**Background:**

The prevalence of hypertension in children is rising globally, with early-onset high blood pressure linked to future cardiovascular risk. Identifying early risk markers beyond obesity and high salt intake is necessary. Although cost-effective indicators of insulin resistance (IR), such as TyG and METS-IR, have been associated with new-onset hypertension in adults, their links with pediatric hypertension, particularly specific phenotypes of hypertension remain unclear.

**Methods:**

12,087 individuals aged 7–17 years from the 2017 China National Nutrition and Health Surveillance of Children and Lactating Women were included. Hypertension was defined as systolic blood pressure (SBP) and/or diastolic blood pressure (DBP) ≥95th percentile for sex, age, and height. Isolated systolic hypertension (ISH), defined as SBP ≥95th and DBP <95th percentile. Isolated diastolic hypertension (IDH), defined as DBP ≥95th and SBP <95th percentile. Systolic-diastolic hypertension (SDH), defined as both SBP and DBP ≥95th percentile. The associations of TyG and METS-IR with hypertension phenotypes were investigated using multivariable logistic regression and restricted cubic spline regression.

**Results:**

TyG and METS-IR were positively associated with hypertension and all its phenotypes after multivariable adjustment. Treated as continuous variables, each 1-unit rise in TyG corresponds to 44%, 47%, and 61% higher chance of ISH, IDH, and SDH, respectively (odds ratio [OR]: 1.44, 95% confidence interval [CI]: 1.31–1.59; OR: 1.47, 95%CI: 1.21–1.79; OR: 1.61, 95%CI: 1.35–1.91); each 1-unit rise in METS-IR corresponds to 10%, 6%, and 12% higher chance of ISH, IDH, and SDH, respectively (OR: 1.10, 95%CI: 1.09–1.12; OR: 1.06, 95%CI: 1.03–1.08; OR: 1.12, 95%CI: 1.10–1.14). Consistent positive associations were observed across different subgroups for ISH and SDH, whereas this association for IDH was not statistically significant in several subgroups (e.g., age ≥12 years, sufficient sleep, daily exercise). TyG and METS-IR exhibited linear dose-response relationships with all hypertension phenotypes (p-nonlinear >0.10).

**Conclusion:**

TyG and METS-IR show strong relationships with three kinds of hypertension phenotypes. They are promising markers that may contribute to the primary prevention of hypertension in pediatric populations.

## Introduction

Hypertension is a leading global risk factor for mortality (1). Since 1990, the prevalence of hypertension among children and adolescents has shown an upward trend, which is expected to persist (2, 3). A 2019 meta-analysis in JAMA Pediatrics reported that global childhood hypertension peaks in pubertal children aged 14– 15 years, with an estimated prevalence of 7.9% in 2015 (3). Alarmingly, evidence indicates that children as young as 11 years can exhibit organ damage due to hypertension (4). Moreover, increased blood pressure (BP) in childhood can be tracked into adulthood, and is associated with elevated BP levels and cardiovascular diseases in late life (5–8). These findings highlight the importance of early prevention and intervention for childhood hypertension. While obesity and high salt intake are well-acknowledged modifiable risk factors for pediatric hypertension (9), most pediatric patients with primary hypertension do not have these factors (10–12), and obese children can still maintain normal BP (13, 14). Identifying additional risk indicators presents more chances for early screening and intervention for pediatric hypertension, reducing future cardiovascular risk.

Insulin resistance (IR) is a significant factor in the pathophysiology of hypertension (15). Recent studies have shown that several non-insulin-based insulin resistance (NI-IR) indices are associated with hypertension and various cardiovascular diseases, serving as independent predictors of cardiovascular mortality (15–18). Among these indices, the triglyceride-glucose index (TyG) and the metabolic score for insulin resistance (METS-IR) have gained much attention, which show a high consistency with IR (18–21). Although the hyperinsulinemic-euglycemic clamp (HEC) is the gold standard for assessing IR, its high cost and technical complexity restrict its application in small-scale studies (18, 22). In contrast, NI-IR indices offer a cost-effective alternative suitable for large epidemiological research (23), and can be used in primary healthcare settings and resource-limited regions (24, 25).

Though previous studies have examined the relationship between NI-IR markers and hypertension (15, 26–28), the majority have focused on adult populations, with limited research on pediatric populations. Moreover, hypertension is a heterogeneous condition, and different phenotypes—including isolated systolic hypertension, isolated diastolic hypertension, and systolic-diastolic hypertension—demonstrate distinct prevalence patterns and clinical implications (29–33). To our knowledge, no studies have investigated the association of TyG and METS-IR with various hypertension phenotypes in underage individuals. Recognizing risk indicators for different hypertension subtypes might offer incremental prevention information and guide targeted interventions, especially in pediatric populations where early intervention and management potentially alleviate the future disease burden of hypertension in the overall population. Thus, this study aims to comprehensively evaluate the associations of TyG and METS-IR with hypertension and its phenotypes in children and adolescents using a relevant dataset.

## Materials and methods

### Data and study subjects

This study utilized data from the 2017 China National Nutrition and Health Surveillance of Children and Lactating Women (34). This surveillance employed a stratified multi-stage cluster random sampling design to select participants. A total of 125 survey sites were systematically selected across 31 provinces in China. Site selection took into account the distribution balance of regional and urban-rural stratification factors, existing work basis and conditions. These 125 monitoring sites were allocated proportionally to four types of regions based on population size: 5 large cities, 57 medium/small cities, 50 rural areas, and 13 impoverished rural areas. In each survey site (city/district/county), two townships or sub-districts were randomly selected, and two villages or neighborhood committees were randomly chosen from each township or street district. For children and adolescents, students from 10 grades were surveyed at each site, including grades 1–6 in two primary schools, grades 7–8 in two junior high schools, and grades 10–11 in one senior high school (the schools were randomly selected from each monitoring site). Considering the academic load and the poor compliance, grade 9 and grade 12 were not included in this surveillance. Subsequently, one class was randomly selected for each grade, and 28 students with equal numbers of males and females were chosen from each selected class. The collected surveillance data is representative at both the provincial and national levels.

Due to limited access to the entire national dataset, we used data from five provinces: Shandong, Jiangsu, Guangdong, Guizhou, and Inner Mongolia, which are geographically located in the East, South, Southwest, and North of China. The initial dataset of the five provinces included 15,673 children and adolescents. As our study focused on participants aged 7 to 17 years, we excluded 866 individuals who fell outside this age range. Additionally, 259 participants lacked laboratory data for calculating TyG and METS-IR, and 193 participants missing variables necessary to determine BP status were excluded. Subsequently, 2,268 individuals were excluded due to missing covariates. A flowchart of the subjects’ exclusion process is presented in **Supplementary Figure 1**. A total of 12,087 participants entered the final analysis. Informed consent was obtained from all participants or their legal guardians.

### Questionnaire surveys, anthropometric measurements, and laboratory tests

The participants received a standardized questionnaire to obtain their demographic, health-related, and lifestyle information. All questionnaires were asked and completed in person by uniformly trained investigators.

Measurements were taken using uniform instruments at each monitoring site. Height was measured in the standing position with shoes removed using a metal TZG-type stadiometer with an accuracy of 0.1 cm. Body weight was measured in the fasted state, with the subjects in underwear and without shoes, using an electronic scale (TANITA, HD-390) that was accurate at 0.01 kg. Waist circumference (WC) was measured horizontally at the midpoint between the inferior edge of the rib cage and the iliac crest along the mid-axillary line in the fasting state using a tape. Systolic blood pressure (SBP) and diastolic blood pressure (DBP) were measured three times at one-minute intervals using an electronic sphygmomanometer (Omron HBP 1300, Tokyo, Japan) in the morning on the left arm (unless otherwise specified). The mean of the two closest readings among the three measurements was taken. The participants were instructed to avoid intense physical activity, eating, or drinking within one hour before the measurement.

Fasting blood samples (6 ml) were collected for biochemical parameters. Fasting plasma glucose (FPG) was measured using the glucokinase method (Roche P800 automatic biochemical analyzer). Triglyceride (TG), total cholesterol (TC), low-density lipoprotein cholesterol (LDL-C), and high-density lipoprotein cholesterol (HDL-C) were measured using a Roche Cobas C701 automatic analyzer.

### Definitions

TyG was calculated using the formula: ln[TG (mg/dL) × FPG (mg/dL)/2] (15). The METS-IR was calculated as ln[(2 × FPG (mg/dL) + TG (mg/dL)) × BMI (kg/m^2^)/ln[HDL-C (mg/dL)] (15).

The current definition of pediatric hypertension is based on the normative distribution of BP in healthy children (6, 35). According to sex, age, and height percentiles, normal blood pressure (NBP) was defined as SBP and DBP < 90th percentile, prehypertension as SBP and/or DBP ≥ 90th and < 95th percentile. Prehypertension is previously referred to as “high normal blood pressure”, a term now replaced by “elevated blood pressure” (EBP) (6), and they are equivalent. Hypertension was defined as SBP and/or DBP ≥ 95th percentile for sex, age, and height (35). To align our definition with the China National Health Industry Standard for children and adolescents, this study refers to SBP and/or DBP ≥ 95th percentile as high blood pressure (HBP) in later text (36). Isolated systolic HBP (ISH) was defined as SBP ≥ 95th and DBP < 95th percentile. Isolated diastolic HBP (IDH) was defined as DBP ≥ 95th and SBP < 95th percentile. Systolic-diastolic HBP (SDH) was defined as both SBP and DBP ≥ 95th percentile.

### Covariates

The covariates included in this study were demographic variables (age, sex, parental education level, residence), health-related and lifestyle variables [abdominal obesity, estimated glomerular filtration rate (eGFR), moderate-vigorous physical activity (MVPA), sleep sufficiency, passive smoking, alcohol intake, unhealthy dietary quality score, and family history of hypertension], and biochemical indicators (serum uric acid, total protein, TC, LDL-C).

Age was calculated by subtracting the date of birth from the survey date, and the full years were taken. Parental education level was categorized into three groups based on the highest level of education attained by both parents: Low—both parents had a primary school education/lower, or one had a primary school education/lower and the other had a secondary school education/diploma; Medium—both parents had a secondary school education/diploma, or one had a primary school education/lower and the other had a bachelor’s degree/higher; High—both parents had a bachelor’s degree/higher, or one had a secondary school education/diploma and the other had a bachelor’s degree/higher.

Abdominal obesity was defined as a WC at or above the 90th percentile for age and sex, determined according to the cutoff points specified in the National Health Industry Standard of China for children and adolescents (37). The eGFR was calculated using the formula recommended by the Chinese guideline for early screening of pediatric chronic kidney disease (38): K × height (cm) × 88.4/serum-creatinine (μmol/L). The constant K is defined as follows: for children aged 2–12 years, K = 0.55; for individuals aged >12 years, K = 0.77 for boys and 0.55 for girls.

Sleep sufficiency was defined based on recommendations by the Ministry of Education of the People’s Republic of China (39), with sleep duration greater than 8 hours for high school students, greater than 9 hours for middle school students, and greater than 10 hours for primary school students. Participants meeting these criteria were classified as “yes” for sufficient sleep, and “no” otherwise. Family history of hypertension was defined as having at least one of the following family members diagnosed with hypertension: father, mother, paternal grandparents, or maternal grandparents.

The unhealthy dietary quality score was calculated based on data from a food frequency questionnaire recording the various foods consumed by participants over the past month and the Chinese Food Guide Pagoda issued by the Chinese Nutrition Society (40). The detailed scoring process for unhealthy dietary quality score can be found in our previous article (41). The remaining covariates were simple self-reports, investigator reports, or laboratory tests.

### Statistical analysis

All the statistical analyses were conducted using R Project for Statistical Computing version 4.2.3 (Vienna, Austria). A two-sided p-value <0.05 was considered statistically significant. Continuous variables were presented as medians and interquartile ranges, whereas categorical variables were reported as frequencies (percentages). Kruskal-Wallis tests, Welch’s ANOVA, and chi-square tests were used to compare variable differences across groups where appropriate. *Post hoc* pairwise comparisons with Bonferroni correction were performed between the NBP group and the EBP group, as well as between the NBP group and the HBP group, yielding a statistically significance threshold of p-value <0.025 (0.05/2). For pairwise comparisons, the Mann-Whitney *U* tests with a Bonferroni correction were applied to continuous variables, while the chi-square tests with a Bonferroni correction were used for categorical variables.

Multinomial logistic regression analyses were performed to evaluate the associations of TyG and METS-IR with different BP groups, with the NBP group as the reference group. The results were expressed as odds ratios (ORs) and 95% confidence intervals (CIs). Multicollinearity was evaluated with the Variance Inflation Factor (VIF), ensuring all variables had VIF values below 5. Restricted cubic spline (RCS) regression was used to explore potential non-linear relationships, with the number of knots set to 3 for smooth curve fitting.

Stratified analysis by age, sex, sleep sufficiency, MVPA, and family history of hypertension was performed to investigate potential heterogeneity across subgroups. Sensitivity analysis was conducted using data after propensity score matching (PSM) with a 1:1 nearest-neighbor matching algorithm and a caliper width of 0.20. The propensity score was estimated using a logistic regression model, and the variables used in calculating the score included age, abdominal obesity, MVPA, sleep sufficiency, alcohol intake, family history of hypertension, parental education level, serum creatinine, total protein, TC, and LDL-C, which were covariates with p<0.05 in baseline table, excluding those used to calculate TyG and METS-IR.

## Results

### General characteristics of children and adolescents

This study analyzed data for 12,087 subjects (50.24% girls and 49.76% boys) aged 7–17 years and their basic characteristics by BP status are presented in **Table 1**. The median age of the participants was 11 years. Compared with the NBP group, both the EBP group and the HBP group had a significantly greater proportion of individuals with abdominal obesity (p<0.025). Furthermore, BMI, total protein, TG, FPG, TyG, and METS-IR levels were significantly higher in both the EBP and HBP groups than in the NBP group (p<0.025). TC and LDL-C in the HBP group were significantly higher than those in the NBP group (p<0.025) but showed no statistically significant differences between EBP and NBP (p>0.025). In contrast, the serum creatinine level was significantly higher in the NBP group than in the other two groups (p<0.025). Additionally, sleep sufficiency, MVPA, alcohol intake, family history of hypertension, and parental education level differed significantly across groups (p<0.05).

**Table 1:** Baseline characteristics of children and adolescents included in this study.

Variables	NBP (n=7804)	EBP (n=1639)	HBP (n=2644)	*P-*value
Age, years	11.0 (9.0, 14.0)	11.0 (9.0, 13.0)	11.0 (9.0, 13.0)	<0.001
Males	3908 (50.08%)	804 (49.05%)	1303 (49.28%)	0.643
BMI, kg/m^2^	17.6 (15.7, 19.9)	18.4 (16.0, 21.1)	18.7 (16.2, 22.1)	<0.001
Abdominal obesity	906 (11.61%)	309 (18.85%)	684 (25.87%)	<0.001
MVPA, days/week	3.0 (1.0, 5.0)	3.0 (1.0, 5.0)	3.0 (1.0, 5.0)	<0.001
Sufficient sleep	2418 (30.98%)	546 (33.31%)	932 (35.25%)	<0.001
Passive smoking, days/week				0.177
almost none	5653 (72.44%)	1204 (73.46%)	1939 (73.34%)	
1-3	1006 (12.89%)	174 (10.62%)	324 (12.25%)	
4-6	329 (4.22%)	68 (4.15%)	102 (3.86%)	
7	816 (10.46%)	193 (11.78%)	279 (10.55%)	
Alcohol intake				<0.001
never drank	6780 (86.88%)	1500 (91.52%)	2422 (91.60%)	
more than 30 days ago	725 (9.29%)	97 (5.92%)	154 (5.82%)	
in the last 30 days	299 (3.83%)	42 (2.56%)	68 (2.57%)	
Unhealthy dietary quality score	6.0 (5.0, 7.0)	6.0 (5.0, 7.0)	6.0 (5.0, 7.0)	0.777
Family history of hypertension	2691 (34.48%)	539 (32.89%)	910 (34.42%)	0.001
Parental education level				<0.001
low	2414 (30.93%)	493 (30.08%)	789 (29.84%)	
medium	4526 (58.00%)	998 (60.89%)	1643 (62.14%)	
high	789 (10.11%)	134 (8.18%)	190 (7.19%)	
unknown	75 (0.96%)	14 (0.85%)	22 (0.83%)	
Rural residence	3381 (43.32%)	739 (45.09%)	1137 (43.00%)	0.359
Serum uric acid, μmol/L	320.6 (269.0, 384.2)	317.0 (267.1, 379.0)	319.0 (267.5, 381.0)	0.279
Serum creatinine, μmol/L	52.0 (45.0, 63.0)	51.0 (44.0, 61.0)	50.0 (43.0, 59.0)	<0.001
eGFR, ml/(min·1.73m^2^)	144.2 (128.5, 160.9)	144.0 (128.6, 160.6)	146.1 (130.8, 162.2)	0.098
Total protein, g/L	75.5 (72.2, 78.9)	76.0 (73.0, 79.5)	76.7 (73.4, 80.0)	<0.001
TG, mg/dL	70.0 (55.8, 92.1)	73.5 (57.6, 94.8)	75.3 (59.3, 99.2)	<0.001
TC, mg/dL	152.4 (134.6, 171.7)	152.7 (135.3, 171.9)	155.1 (136.9, 175.6)	<0.001
LDL-C, mg/dL	80.0 (66.1, 95.1)	80.8 (66.5, 96.7)	82.4 (66.9, 98.7)	<0.001
HDL-C, mg/dL	56.1 (48.3, 65.0)	56.5 (48.7, 65.0)	56.8 (48.7, 66.5)	0.074
FPG, mg/dL	93.2 (87.7, 98.3)	94.1 (88.3, 99.3)	94.3 (88.7, 99.9)	<0.001
TyG	8.1 (7.8, 8.4)	8.1 (7.9, 8.4)	8.2 (7.9, 8.5)	<0.001
METS-IR	24.3 (21.3, 28.1)	25.2 (21.7, 29.9)	25.8 (22.1, 31.4)	<0.001

*NBP*, normal blood pressure; *EBP*, elevated blood pressure; *HBP*, high blood pressure; *BMI*, body mass index; *MVPA*, moderate-vigorous physical activity; *eGFR*, estimated glomerular filtration rate; *TG*, triglyceride; *TC*, total cholesterol; *LDL-C*, low-density lipoprotein cholesterol; *HDL-C*, high-density lipoprotein cholesterol; *FPG*, fasting plasma glucose; *TyG*, triglyceride-glucose index; *METS-IR*, metabolic score for insulin resistance.

*P-*values were based on Kruskal-Wallis tests, Welch’s ANOVA, or Chi-square tests, as appropriate. Data were expressed as median (interquartile range) or number (percentage).

### Associations of TyG and METS-IR with elevated blood pressure and high blood pressure

**Table 2** presents the associations of TyG and METS-IR with EBP and HBP. TyG and METS-IR were divided into quartiles, with the lowest quartile (Q1) as the reference group. In the fully adjusted Model 2, the ORs for EBP and HBP was 1.2-fold and 1.4-fold higher in the highest quartile groups of TyG (OR: 1.24, 95%CI: 1.06–1.45, p<0.01; OR: 1.44, 95%CI: 1.26–1.65, p<0.001) and 2.3-fold and 3.1-fold higher in the highest quartile groups of METS-IR (OR: 2.32, 95%CI: 1.88–2.86, p<0.001; OR: 3.06, 95%CI: 2.56–3.66, p<0.001), compared with the lowest quartile groups of TyG and METS-IR. Higher quartiles of METS-IR were more strongly linked to the presence of EBP and HBP than the corresponding quartiles of TyG. **Supplementary Figure 2** illustrated that TyG and METS-IR had linear relationships with EBP and HBP (p for non-linear >0.10).

**Table 2:** Association of insulin resistance index with elevated blood pressure and high blood pressure (*OR* (95%*CI*)).

Insulin resistance indices		Elevated blood pressure		High blood pressure	
		Model 1	Model 2	Model 1	Model 2
TyG	Q1 (≤7.85)	1 (reference group)	1 (reference group)	1 (reference group)	1 (reference group)
	Q2 (7.85-8.11)	1.07 (0.92, 1.25)	1.04 (0.89, 1.22)	1.16 (1.01, 1.32)*	1.09 (0.95, 1.24)
	Q3 (8.11-8.39)	1.30 (1.12, 1.52)‡	1.22 (1.04, 1.43)*	1.58 (1.39, 1.80)‡	1.39 (1.22, 1.59)‡
	Q4 (≥8.39)	1.41 (1.21, 1.64)‡	1.24 (1.06, 1.45)†	1.88 (1.65, 2.13)‡	1.44 (1.26, 1.65)‡
	continuous	1.43 (1.25, 1.63)‡	1.27 (1.15, 1.40)‡	1.89 (1.69, 2.11)‡	1.48 (1.35, 1.61)‡
METS-IR	Q1 (≤21.53)	1 (reference group)	1 (reference group)	1 (reference group)	1 (reference group)
	Q2 (21.53-24.71)	1.25 (1.07, 1.47)†	1.24 (1.06, 1.46)†	1.42 (1.24, 1.62)‡	1.37 (1.20, 1.57)‡
	Q3 (24.72-28.97)	1.49 (1.26, 1.77)‡	1.49 (1.24, 1.78)‡	1.78 (1.54, 2.06)‡	1.65 (1.42, 1.92)‡
	Q4 (≥28.97)	2.44 (2.05, 2.90)‡	2.32 (1.88, 2.86)‡	3.87 (3.35, 4.47)‡	3.06 (2.56, 3.66)‡
	continuous	1.06 (1.05, 1.07)‡	1.07 (1.06, 1.08)‡	1.10 (1.09, 1.11)‡	1.10 (1.09, 1.11)‡

Model 1: adjusted for age, sex; Model 2: adjusted for age, sex, abdominal obesity, moderate-vigorous physical activity, sleep sufficiency, passive smoking, alcohol intake, parental education level, unhealthy dietary quality score, family history of hypertension, residence, serum uric acid, estimated glomerular filtration rate, total protein, total cholesterol, and low-density lipoprotein cholesterol.

*OR*, odds ratio; *CI*, confidence interval; *TyG*, triglyceride-glucose index; *METS-IR*, metabolic score for insulin resistance.

*indicating *P* < 0.05, †indicating *P* < 0.01, ‡indicating *P* < 0.001.

### Associations of TyG and METS-IR with different high blood pressure phenotypes

**Figure 1:**
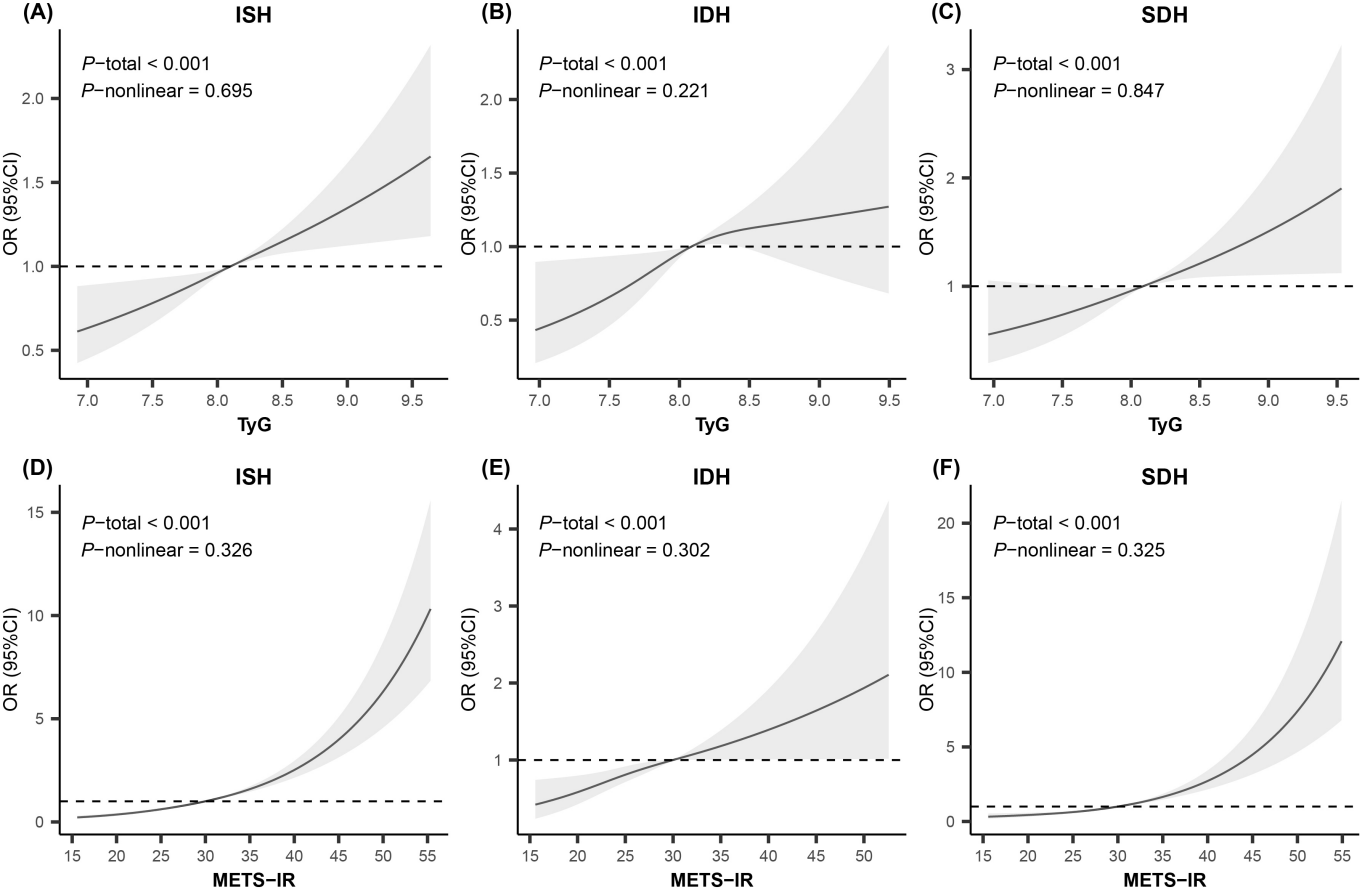
Restricted cubic spline analysis for the relationship of insulin resistance indices with high blood pressure phenotypes. Knots were set to 3 for smooth curve fitting. Adjusted for age, sex, abdominal obesity, moderate-vigorous physical activity, sleep sufficiency, passive smoking, alcohol intake, parental education level, unhealthy dietary quality score, family history of hypertension, residence, serum uric acid, estimated glomerular filtration rate, total protein, total cholesterol, and low-density lipoprotein cholesterol. *OR*, odds ratio; *CI*, confidence interval; *ISH*, isolated systolic high blood pressure; *IDH*, isolated diastolic high blood pressure; *SDH*, systolic-diastolic high blood pressure; *TyG*, triglyceride-glucose index; *METS-IR*, metabolic score for insulin resistance.

**Table 3** shows the associations of these two NI-IR indices with various HBP subtypes. Both Model 1 and Model 2 indicated that the third quartile (Q3) and the highest quartile (Q4) of TyG and METS-IR were positively associated with all HBP phenotypes, as were continuous TyG and METS-IR. After adjusting for potential confounders in Model 2, the ORs for ISH, IDH and SDH in the highest TyG quartile groups compared with the lowest groups were 1.39 (95%CI: 1.19–1.63, p<0.001), 1.56 (95%CI: 1.13–2.16, p<0.01), and 1.53 (95%CI: 1.16–2.01, p<0.01), respectively. For METS-IR, the ORs for ISH, IDH and SDH were 3.5-fold, 1.9-fold and 3.0-fold higher in the highest quartile groups than in the lowest quartile groups (OR: 3.46, 95%CI: 2.81–4.26, p<0.001; OR: 1.85, 95%CI: 1.23–2.80, p<0.01; OR: 3.02, 95%CI: 2.12–4.32, p<0.001). The RCS analysis indicated linear relationships between TyG, METS-IR, and all HBP phenotypes (p for non-linear >0.10), as illustrated in **Figure 1**.

**Table 3:** Association of insulin resistance index with different high blood pressure phenotypes (*OR* (95%*CI*)).

Insulin resistance indices		ISH		IDH		SDH	
		Model 1	Model 2	Model 1	Model 2	Model 1	Model 2
TyG	Q1 (≤7.85)	1 (reference group)	1 (reference group)	1 (reference group)	1 (reference group)	1 (reference group)	1 (reference group)
	Q2 (7.85-8.11)	1.15 (0.99, 1.35)	1.08 (0.92, 1.27)	1.35 (0.98, 1.85)	1.25 (0.91, 1.72)	1.04 (0.78, 1.38)	0.96 (0.72, 1.28)
	Q3 (8.11-8.39)	1.54 (1.32, 1.79)‡	1.34 (1.14, 1.56)‡	1.89 (1.39, 2.56)‡	1.71 (1.25, 2.33)‡	1.55 (1.18, 2.02)†	1.37 (1.04, 1.80)*
	Q4 (≥8.39)	1.86 (1.60, 2.16)‡	1.39 (1.19, 1.63)‡	1.82 (1.33, 2.49)‡	1.56 (1.13, 2.16)†	1.98 (1.52, 2.57)‡	1.53 (1.16, 2.01)†
	continuous	1.90 (1.67, 2.15)‡	1.44 (1.31, 1.59)‡	1.68 (1.30, 2.16)‡	1.47 (1.21, 1.79)‡	2.03 (1.63, 2.54)‡	1.61 (1.35, 1.91)‡
METS-IR	Q1 (≤21.53)	1 (reference group)	1 (reference group)	1 (reference group)	1 (reference group)	1 (reference group)	1 (reference group)
	Q2 (21.53-24.71)	1.50 (1.28, 1.77)‡	1.44 (1.23, 1.70)‡	1.21 (0.90, 1.62)	1.20 (0.89, 1.62)	1.35 (1.03, 1.76)*	1.32 (1.00, 1.73)*
	Q3 (24.72-28.97)	1.89 (1.59, 2.25)‡	1.72 (1.43, 2.06)‡	1.54 (1.12, 2.11)†	1.58 (1.14, 2.20)†	1.67 (1.25, 2.25)‡	1.62 (1.19, 2.21)†
	Q4 (≥28.97)	4.63 (3.91, 5.48)‡	3.46 (2.81, 4.26)‡	1.81 (1.29, 2.54)†	1.85 (1.23, 2.80)†	3.51 (2.64, 4.67)‡	3.02 (2.12, 4.32)‡
	continuous	1.11 (1.10, 1.12)‡	1.10 (1.09, 1.12)‡	1.04 (1.02, 1.06)‡	1.06 (1.03, 1.08)‡	1.10 (1.09, 1.12)‡	1.12 (1.10, 1.14)‡

Model 1: adjusted for age, sex; Model 2: adjusted for age, sex, abdominal obesity, moderate-vigorous physical activity, sleep sufficiency, passive smoking, alcohol intake, parental education level, unhealthy dietary quality score, family history of hypertension, residence, serum uric acid, estimated glomerular filtration rate, total protein, total cholesterol, and low-density lipoprotein cholesterol.

*OR*, odds ratio; *CI*, confidence interval; *ISH*, isolated systolic high blood pressure; *IDH*, isolated diastolic high blood pressure; *SDH*, systolic-diastolic high blood pressure; *TyG*, triglyceride-glucose index; *METS-IR*, metabolic score for insulin resistance.

*indicating *P* < 0.05, †indicating *P* < 0.01, ‡indicating *P* < 0.001.

### Stratified and sensitivity analyses

**Figure 2:**
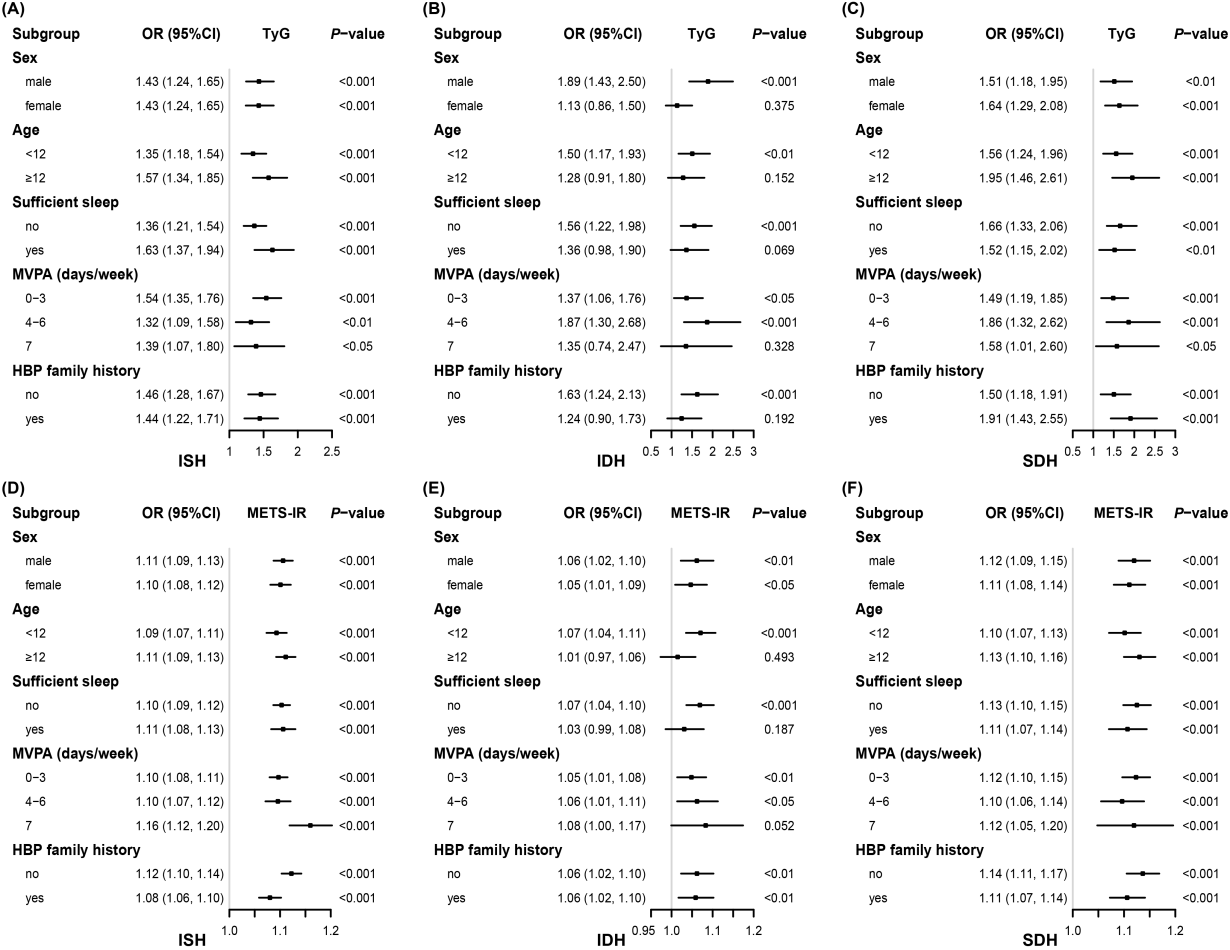
Associations between insulin resistance indices and high blood pressure phenotypes stratified by different factors. Adjusted for, if not stratified, age, sex, abdominal obesity, moderate-vigorous physical activity, sleep sufficiency, passive smoking, alcohol intake, parental education level, unhealthy dietary quality score, family history of hypertension, residence, serum uric acid, estimated glomerular filtration rate, total protein, total cholesterol, and low-density lipoprotein cholesterol. *OR*, odds ratio; *CI*, confidence interval; *TyG*, triglyceride-glucose index; *METS-IR*, metabolic score for insulin resistance; *ISH*, isolated systolic high blood pressure; *IDH*, isolated diastolic high blood pressure; *SDH*, systolic-diastolic high blood pressure; *HBP*, high blood pressure; *MVPA*, moderate-vigorous physical activity.

**Figure 2** consistently demonstrates a significantly positive association of TyG and METS-IR with ISH across all strata. The results for SDH are similar. However, for IDH, in the strata where individuals were aged 12 years and older, had sufficient sleep, and engaged in daily MVPA, neither the TyG nor the METS-IR showed a statistically significant association with IDH. The subgroup sample sizes are detailed in **Supplementary Table 1**.

Sensitivity analysis was conducted using data after PSM. The results of matching and the characteristics of the new sample are presented in **Supplementary Table 2**. Utilizing the new dataset, we re-explored the associations between TyG, METS-IR, and different HBP subtypes, and it remained consistent with our previous findings, suggesting that TyG and METS-IR may link to increased risk of all kinds of HBP phenotypes in children and adolescents (**Supplementary Table 3**).

## Discussion

To our knowledge, this is the first study to examine the associations of TyG and METS-IR with various HBP phenotypes in children and adolescents. It revealed that TyG and METS-IR were positively associated with HBP and its three phenotypes, with particularly robust associations observed for ISH and SDH. Linear dose-response relationships existed between these two indices and all HBP phenotypes. The findings imply that TyG and METS-IR may be significant predictors of HBP in children and adolescents, contributing to primary prevention strategies.

IR plays a critical role in the pathophysiology of HBP, contributing to increasing BP through multiple proposed mechanisms, including enhanced tissue angiotensin II and aldosterone activities, increased sympathetic nervous system activity, and oxidative stress (42–44). A study in 2010 provided the basis for accepting the TyG as a surrogate tool for assessing IR (23). TyG performs as well as, or better than, the homeostasis model assessment of IR (HOMA-IR), which requires insulin measurement (18, 20). Previous cohort studies have affirmed that high TyG levels are associated with a greater risk of new-onset hypertension (27, 45–47). However, those studies focused only on adults. Only a cross-sectional study conducted on individuals 6–15 years in Mexico stated that elevated TyG was significantly related to pediatric hypertension (48), but it did not explore the dose-response relationship nor further examine the association between TyG and specific subtypes of hypertension. Introduced in 2018, METS-IR offers a new reliable non-insulin-based approach to assess IR (21). It shows good agreement with HEC and frequently sampled intravenous glucose tolerance tests (21), outperforming TyG in predicting IR (19). Up to now, there have been no studies on the association of METS-IR with hypertension and its phenotypes in pediatric populations. Concerning adults, a recent meta-analysis that included 8 cohort studies and 305,341 individuals demonstrated that elevated METS-IR is tied to hypertension in the general adult population (49). Our findings align with most existing studies investigating the association of TyG and METS-IR with hypertension.

However, it is important to note that both indices are non-insulin-based surrogate markers for IR. We compared our results with studies examining the relationship between insulin-based indices and hypertension. Two commonly used insulin-based indices for IR are the Homeostatic Model Assessment of Insulin Resistance (HOMA-IR) and the Quantitative Insulin Sensitivity Check Index (QUICKI). Vizzuso et al. recruited 70 obese White children and adolescents aged 7–16 years and used ambulatory blood pressure monitoring to examine the relationship between IR and hypertension. They found that both HOMA-IR and QUICKI were associated with the presence of hypertension and were thus helpful in identifying hypertensive obese pediatric patients (50). Similarly, a study based on the Jackson Heart cohort by Kaze et al. reported that among blacks both HOMA-IR and QUICKI were associated with the risk of blood pressure progression and incident hypertension (51). Moreover, a systematic review encompassing 38 studies demonstrated that higher HOMA-IR values significantly increased the risk of developing hypertension, indicating its potential as a predictor (52). The direction of our findings is consistent with the above evidence, supporting the idea that IR indices may help recognize individuals at risk of hypertension. Other evidence has shown that both HOMA-IR and QUICKI are correlated with SBP and DBP in children and adolescents (53, 54), which suggests their relevance to different hypertension subtypes in this age group. However, we did not find studies that explored the associations between these two insulin-based indices and specific hypertension phenotypes in pediatric populations.

Clinical impacts of different hypertension phenotypes vary. Compared with other phenotypes, ISH poses a higher risk of stroke and coronary heart disease (29, 30). SDH is associated with increased cardiovascular risk but is less prevalent (30). While IDH is recognized as a risk factor for cardiovascular disease, its impact on incident cardiovascular outcomes is questioned by some studies (32, 55). To date, relatively few studies have investigated the relationships of TyG and METS-IR with detailed hypertension subtypes, and all of them pay attention to adults. In China, elevated TyG levels have been significantly associated with ISH in middle-aged and elderly adults (56), and with increased risks of IDH and SDH (57). Additionally, a cohort study among young military adults observed that TyG was associated with the risk of IDH and SDH, whereas METS-IR was linked only to IDH (15).

In our study, TyG and METS-IR showed robust positive associations with ISH and SDH. Although significant association with IDH was observed in the overall sample, the association was not detected in certain subgroups. Specifically, neither TyG nor METS-IR was significantly associated with IDH among participants aged ≥ 12 years, those with sufficient sleep, or those engaging in daily MVPA. Most notably, the simultaneously non-significant association of TyG and METS-IR was observed exclusively in specific subgroups of IDH. This possibly involves the distinct pathophysiological mechanisms underlying each HBP phenotype. Generally, it is thought that ISH may result from arterial stiffening, whereas IDH is related to an increase in peripheral vascular resistance (57–60). The association of both indices with ISH was robust across all subgroups, potentially indicating that in children the relationship between IR and arterial stiffness is almost unaffected by those stratification variables. SDH may benefit from the contribution of elevated SBP, and thus the association of the two indices with SDH was similar to that of ISH in all subgroups. However, these stratification factors might influence peripheral vascular resistance. Studies indicate that healthy lifestyle behaviors, such as adequate sleep and regular exercise, can reduce peripheral resistance (61–64), which may help mitigate the effects of IR on IDH. In addition, IDH is age-dependent and becomes less prevalent with age (31, 57), but the disappearance of the association of both indices with IDH in the participants ≥ 12 years here is unlikely to be attributable to aging, as this association can still be observed in adult populations (15, 57). We speculate that hormonal changes during puberty affect IR (65), obscuring the association between IR indices and IDH. Another possibility is insufficient statistical power in certain subgroups, such as daily MVPA, due to the inadequate number of IDH cases (**Supplementary Table 1**). To verify the robustness of these non-significant associations, additional stratified analysis was performed using two approaches: adjusting for different sets of covariates and utilizing PSM-derived data. Interestingly, the results remained consistent (**Supplementary Figure 3**). Future studies are warranted to confirm these findings and to determine whether they reflect true heterogeneity or arise from limited cases.

Our results on the linear dose-response relationship between two IR indices and hypertension are in accordance with the majority of findings on Asian adults (45, 66–70). However, some studies identified non-linear relationships between METS-IR and hypertension in American adults (25, 71). This discrepancy may be due to ethnic differences, as research suggests that insulin sensitivity varies by ethnicity. Specifically, East Asians exhibit higher insulin sensitivity and a lower insulin response than Africans and Caucasians (72).

We also observed that in Model 2, among the three hypertensive subtypes, the ORs for TyG in the higher quartiles (Q2, Q3, and Q4) were greatest in IDH, whereas those for METS-IR were lowest in IDH. The results of the sensitivity analysis were consistent (**Supplementary Table 3**). It may indicate that in pediatric populations, TyG has a greater advantage in identifying the risk of IDH than ISH, whereas METS-IR is more effective in identifying ISH and SDH. Some possible explanations are made. Calculated using FPG and TG, TyG primarily reflects glycolipid metabolic disorders, which can trigger oxidative stress (73). Oxidative stress, in turn, damages microvascular endothelial cells and reduces nitric oxide production and bioavailability, ultimately leading to microvascular dysfunction (74). In addition, the elevated FPG and abnormal lipid panel may enhance blood viscosity by increasing the rheological component of peripheral resistance, contributing to increased DBP (57). Since IDH is primarily associated with increased peripheral vascular resistance and lesions of arterioles (57, 59), the aforementioned pathophysiological mechanisms may underlie the stronger association between high TyG levels and IDH than ISH. The components of METS-IR include BMI, thus it takes into account obesity in addition to reflecting glycolipid metabolic disorders compared with TyG. METS-IR itself has a significant correlation with visceral fat (21), and the relationship between visceral adiposity and arterial stiffness has been demonstrated (75, 76). Visceral adiposity is often accompanied by abnormal secretion of pro-inflammatory factors (e.g., tumor necrosis factor alpha, interleukin 6) by adipocytes and increased free fatty acids, which promote endothelial dysfunction and vascular stiffness (71). Further, it seems that it is SBP but not DBP more correlated with visceral adiposity (77, 78). Therefore, the additional consideration of adiposity might be the cause of why METS-IR is more strongly associated with ISH and SDH than IDH. Differences in the superiority of TyG and METS-IR for the identification of various hypertension subtypes in children and adolescents may exist.

Longitudinal follow-up of children and adolescents with elevated METS-IR and TyG values is warranted to assess their future incidence of hypertension, validating the predictive capacity of these two NI-IR indices for new-onset hypertension. This may provide valuable tools for the early identification of hypertension risk in children and help develop primary prevention strategies to reduce the future burden of hypertension in the whole population.

### Strengths and limitations

Several strengths can be attributed to this study. Our surveillance data are representative at the provincial level and are collected with rigorous quality assurance and control. We have examined the multiplicative interaction between the province and the IR indices on hypertension prevalence and observed that the interaction was not statistically significant (p for interaction >0.05), which enhances the national generalization of our findings. In addition, this study extends the evidence on this research topic from adults to pediatric populations, thereby contributing to the validation of IR index in children. However, we acknowledge that the present study has several limitations. First, although BP was measured three times for each participant, all readings were obtained during a single visit, an unavoidable constraint in epidemiological studies, which may overestimate the prevalence of hypertension (79). Evaluating BP on three or more separate occasions is helpful, thereby reducing measurement variability. Second, despite extensive efforts to adjust for potential confounders, including demographic information, family history, health-related behaviors and status, diet, and kidney health, residual confounding from unmeasured or unknown factors, such as medication use, may still influence the results. Third, since the study population consisted solely of Chinese minors, the applicability of these findings to other ethnicities remains uncertain. Given that the relation between IR and BP differs among racial groups (80), we propose further research among diverse pediatric ethnic populations to improve the global generalizability of these observations.

Finally, the cross-sectional design of this study limits causal inference, as it collects data on exposure and outcome at a single point in time. Consequently, it precludes establishing the temporal sequence of events—we cannot determine whether IR preceded BP elevation, vice versa, or whether they developed concurrently. This ambiguity poses challenges: reverse causality remains a possible alternative explanation, and the dynamic interplay between IR and BP over time cannot be tracked. This limitation could not be overcome through statistical methods. Therefore, the associations reported in this study should be interpreted with caution and not as direct evidence of causality. To address this, prospective cohort studies with repeated measurements of exposure and BP over time are essential.

## Conclusion

TyG and METS-IR are positively associated with HBP and its three phenotypes in children and adolescents, exhibiting linear relationships. These associations were particularly robust for ISH and SDH, but less consistent for IDH in certain subgroups (age ≥12 years, sufficient sleep, daily MVPA). This may indicate the modification effect of puberty and healthy lifestyles in the impact of IR on IDH. Maintaining relatively low levels of TyG and METS-IR might reduce the risk of developing hypertension in children. In summary, METS-IR and TyG hold potential as useful supplemental indicators for identifying children at high risk for hypertension and for informing targeted management strategies based on their levels, but further longitudinal studies are warranted.

## Data Availability

The data analyzed in this study is subject to the following licenses/restrictions: The datasets generated and/or analyzed during the current study are not publicly available for ethical and privacy reasons but are available from the corresponding author upon reasonable request. Requests to access these datasets should be directed to Xiaomei Dong (e-mail: ntydxm@126.com).

## Supplementary material

Supplementary Table 1 The cases of different high blood pressure phenotypes in each subgroup. *ISH*, isolated systolic high blood pressure; *IDH*, isolated diastolic high blood pressure; *SDH*, systolic-diastolic high blood pressure; *MVPA*, moderate-vigorous physical activity.

Supplementary Table 2 Information of covariates after propensity score matching. *NBP*, normal blood pressure; *HBP*, high blood pressure; *SMD*, standardized mean difference; *MVPA*, moderate-vigorous physical activity; *eGFR*, estimated glomerular filtration rate; *TC*, total cholesterol; *LDL-C*, low-density lipoprotein cholesterol. *P-*values were based on Mann-Whitney U tests or Chi-square tests, as appropriate. Data were presented as median (interquartile) or number (percentage). *Variables used in calculating the propensity score. ^†^An *SMD* < 0.1 indicates a relatively good balance.

Supplementary Table 3 Associations between insulin resistance indices and high blood pressure phenotypes after propensity score matchingAdjusted for age, sex, abdominal obesity, moderate-vigorous physical activity, sleep sufficiency, passive smoking, alcohol intake, parental education level, unhealthy dietary quality score, family history of hypertension, residence, serum uric acid, estimated glomerular filtration rate, total protein, total cholesterol, and low-density lipoprotein cholesterol. *ISH*, isolated systolic high blood pressure; *IDH*, isolated diastolic high blood pressure; *SDH*, systolic-diastolic high blood pressure; *OR*, odds ratio; *CI*, confidence interval; *TyG*, triglyceride-glucose index; *METS-IR*, metabolic score for insulin resistance. * indicating *P* < 0.05, ^†^ indicating *P* < 0.01, ^‡^ indicating *P* < 0.001.

Supplementary Figure 1 Flowchart of inclusion and exclusion. *Participants were from five provinces (Shandong, Jiangsu, Guangdong, Guizhou, Inner Mongolia) within the Surveillance coverage.

Supplementary Figure 2 Restricted cubic spline analysis for the relationship of insulin resistance indices with elevated blood pressure and high blood pressure. Knots were set to 3 for smooth curve fitting. Adjusted for age, sex, abdominal obesity, moderate-vigorous physical activity, sleep sufficiency, passive smoking, alcohol intake, parental education level, unhealthy dietary quality score, family history of hypertension, residence, serum uric acid, estimated glomerular filtration rate, total protein, total cholesterol, and low-density lipoprotein cholesterol. *OR*, odds ratio; *CI*, confidence interval; *EBP*, elevated blood pressure; *HBP*, high blood pressure; *TyG*, triglyceride-glucose index; *METS-IR*, metabolic score for insulin resistance.

Supplementary Figure 3 Association between two insulin resistance indices and IDH stratified by different factors. **(A)** and **(B)** were adjusted for, if not stratified, age, sex, and abdominal obesity, using the entire dataset. **(C)** and **(D)** were adjusted for, if not stratified, age, sex, abdominal obesity, moderate-vigorous physical activity, sleep sufficiency, passive smoking, alcohol intake, parental education level, unhealthy dietary quality score, family history of hypertension, residence, serum uric acid, estimated glomerular filtration rate, total protein, and total cholesterol, using the dataset after propensity score matching. *OR*, odds ratio; *CI*, confidence interval; *HBP*, high blood pressure; *IDH*, isolated diastolic high blood pressure; *TyG*, triglyceride-glucose index; *METS-IR*, metabolic score for insulin resistance.
