# Supplementary material for: Associations of triglyceride-glucose index and metabolic score for insulin resistance with various hypertension phenotypes in children and adolescents: results from the 2017 China nutrition and health surveillance
Source: Front Endocrinol (Lausanne). 2025 Jul 24;16:1595097. doi: 10.3389/fendo.2025.1595097 (PMC12328147; doi:10.3389/fendo.2025.1595097)
Supplement: Supplementary Table 1 — The cases of different high blood pressure phenotypes in each subgroup. ISH, isolated systolic high blood pressure; IDH, isolated diastolic high blood pressure; SDH, systolic-diastolic high blood pressure; MVPA, moderate-vigorous physical activity. [file DataSheet1.docx]

**Supplemental file**

**Supplementary Table 1.** The cases of different high blood pressure phenotypes in each subgroup.

**Supplementary Table 2.** Information of covariates after propensity score matching.

**Supplementary Table 3.** Associations between insulin resistance indices and high blood pressure phenotypes after propensity score matching.

**Supplementary Figure 1.** Flowchart of inclusion and exclusion.

**Supplementary Figure 2.** Restricted cubic spline analysis for the relationship of insulin resistance indices with elevated blood pressure and high blood pressure.

**Supplementary Figure 3.** Association between two insulin resistance indices and IDH stratified by different factors.

**Supplementary Table 1.** The cases of different high blood pressure phenotypes in each subgroup

| Subgroup | ISH | |  | IDH | |  | SDH | |
| --- | --- | --- | --- | --- | --- | --- | --- | --- |
|  | cases/total | cases(%) |  | cases/total | cases(%) |  | cases/total | cases(%) |
| Male | 897/6015 | 14.90% |  | 179/6015 | 3.00% |  | 227/6015 | 3.80% |
| Female | 879/6072 | 14.50% |  | 198/6072 | 3.30% |  | 264/6072 | 4.30% |
| Age (<12) | 1005/6403 | 15.70% |  | 239/6403 | 3.70% |  | 299/6403 | 4.70% |
| Age (≥12) | 771/5684 | 13.60% |  | 138/5684 | 2.40% |  | 192/5684 | 3.40% |
| Sufficient sleep (no) | 1173/8191 | 14.30% |  | 239/8191 | 2.90% |  | 300/8191 | 3.70% |
| Sufficient sleep (yes) | 603/3896 | 15.50% |  | 138/3896 | 3.50% |  | 191/3896 | 4.90% |
| MVPA (0-3) | 996/6555 | 15.20% |  | 226/6555 | 3.40% |  | 307/6555 | 4.70% |
| MVPA (4-6) | 531/3738 | 14.20% |  | 112/3738 | 3.00% |  | 129/3738 | 3.50% |
| MVPA (7) | 249/1794 | 13.90% |  | 39/1794 | 2.20% |  | 55/1794 | 3.10% |
| Family history of hypertension (no) | 965/6304 | 15.30% |  | 198/6304 | 3.10% |  | 257/6304 | 4.10% |
| Family history of hypertension (yes) | 605/4140 | 14.60% |  | 133/4140 | 3.20% |  | 172/4140 | 4.20% |

*ISH*, isolated systolic high blood pressure; *IDH*, isolated diastolic high blood pressure; *SDH*, systolic-diastolic high blood pressure; *MVPA*, moderate-vigorous physical activity.

**Supplementary Table 2.** Information of covariates after propensity score matching

| Variables | NBP (n=2641) | HBP (n=2641) | *P-*value | *SMD*† |
| --- | --- | --- | --- | --- |
| Age, years* | 11.0 (9.0, 13.0) | 11.0 (9.0, 13.0) | 0.935 | 0.001 |
| Males | 1237 (46.84%) | 1300 (49.22%) | 0.083 | 0.048 |
| Abdominal obesity* | 664 (25.14%) | 681 (25.79%) | 0.591 | 0.015 |
| MVPA, days/week* | 3.0 (1.0, 5.0) | 3.0 (1.0, 5.0) | 0.593 | 0.038 |
| Sufficient sleep* | 932 (35.29%) | 930 (35.21%) | 0.954 | 0.002 |
| Passive smoking, days/week |  |  | 0.731 | 0.031 |
| almost none | 1972 (74.67%) | 1936 (73.31%) |  |  |
| 1-3 | 306 (11.59%) | 324 (12.27%) |  |  |
| 4-6 | 96 (3.63%) | 102 (3.86%) |  |  |
| 7 | 267 (10.11%) | 279 (10.56%) |  |  |
| Alcohol intake* |  |  | 0.284 | 0.044 |
| never drank | 2438 (92.31%) | 2419 (91.59%) |  |  |
| more than 30 days ago | 152 (5.76%) | 154 (5.83%) |  |  |
| in the last 30 days | 51 (1.93%) | 68 (2.57%) |  |  |
| Unhealthy dietary quality score | 6.0 (5.0, 7.0) | 6.0 (5.0, 7.0) | 0.750 | 0.012 |
| Family history of hypertension* | 912 (34.53%) | 910 (34.46%) | 0.971 | 0.007 |
| Parental education level* |  |  | 0.942 | 0.017 |
| low | 776 (29.38%) | 789 (29.88%) |  |  |
| medium | 1644 (62.25%) | 1640 (62.10%) |  |  |
| high | 200 (7.57%) | 190 (7.19%) |  |  |
| unknown | 21 (0.80%) | 22 (0.83%) |  |  |
| Rural residence | 1155 (43.73%) | 1137 (43.05%) | 0.617 | 0.014 |
| Serum uric acid, μmol/L | 315.0 (264.0, 376.0) | 319.0 (267.6, 381.0) | 0.111 | 0.056 |
| Serum creatinine, μmol/L* | 50.0 (43.0, 59.0) | 50.0 (43.0, 59.0) | 0.704 | 0.006 |
| eGFR, ml/(min·1.73m^2^) | 145.8 (130.5, 161.8) | 146.1 (130.8, 162.2) | 0.752 | 0.005 |
| Total protein, g/L* | 76.5 (73.3, 80.0) | 76.7 (73.4, 80.0) | 0.850 | 0.01 |
| TC, mg/dL* | 155.1 (137.7, 174.4) | 155.1 (136.9, 175.6) | 0.752 | 0.004 |
| LDL-C, mg/dL* | 82.4 (67.7, 97.8) | 82.4 (66.9, 98.6) | 0.812 | 0.004 |

*NBP*, normal blood pressure; *HBP*, high blood pressure; *SMD*, standardized mean difference; *MVPA*, moderate-vigorous physical activity; *eGFR*, estimated glomerular filtration rate; *TC*, total cholesterol; *LDL-C*, low-density lipoprotein cholesterol.

*P-*values were based on Mann-Whitney U tests or Chi-square tests, as appropriate. Data were presented as median (interquartile) or number (percentage).

*Variables used in calculating the propensity score.

†An *SMD* < 0.1 indicates a relatively good balance.

**Supplementary Table 3.** Associations between insulin resistance indices and high blood pressure phenotypes after propensity score matching

| Insulin resistance indices | | ISH |  | IDH |  | SDH |
| --- | --- | --- | --- | --- | --- | --- |
|  |  | *OR* (95%*CI*) |  | *OR* (95%*CI*) |  | *OR* (95%*CI*) |
| TyG | Q1 (≤7.85) | 1 (reference group) |  | 1 (reference group) |  | 1 (reference group) |
|  | Q2 (7.85-8.11) | 1.04 (0.87, 1.25) |  | 1.21 (0.86, 1.69) |  | 0.93 (0.68, 1.25) |
|  | Q3 (8.11-8.39) | 1.28 (1.07, 1.53)† |  | 1.65 (1.19, 2.28)† |  | 1.30 (0.97, 1.73) |
|  | Q4 (≥8.39) | 1.33 (1.11, 1.60)† |  | 1.49 (1.06, 2.09)* |  | 1.47 (1.10, 1.96)† |
|  | continuous | 1.42 (1.26, 1.60)‡ |  | 1.45 (1.18, 1.79)‡ |  | 1.59 (1.33, 1.92)‡ |
| METS-IR | Q1 (≤21.53) | 1 (reference group) |  | 1 (reference group) |  | 1 (reference group) |
|  | Q2 (21.53-24.71) | 1.38 (1.15, 1.67)‡ |  | 1.17 (0.86, 1.59) |  | 1.25 (0.94, 1.67) |
|  | Q3 (24.72-28.97) | 1.63 (1.33, 2.01)‡ |  | 1.51 (1.07, 2.14)* |  | 1.54 (1.11, 2.13)† |
|  | Q4 (≥28.97) | 3.37 (2.63, 4.32)‡ |  | 1.80 (1.16, 2.79)† |  | 2.90 (1.98, 4.26)‡ |
|  | continuous | 1.10 (1.08, 1.11)‡ |  | 1.05 (1.02, 1.08)‡ |  | 1.11 (1.09, 1.14)‡ |

Adjusted for age, sex, abdominal obesity, moderate-vigorous physical activity, sleep sufficiency, passive smoking, alcohol intake, parental education level, unhealthy dietary quality score, family history of hypertension, residence, serum uric acid, estimated glomerular filtration rate, total protein, total cholesterol, and low-density lipoprotein cholesterol.

*ISH*, isolated systolic high blood pressure; *IDH*, isolated diastolic high blood pressure; *SDH*, systolic-diastolic high blood pressure; *OR*, odds ratio; *CI*, confidence interval; *TyG*, triglyceride-glucose index; *METS-IR*, metabolic score for insulin resistance.

* indicating *P* < 0.05, † indicating *P* < 0.01, ‡ indicating *P* < 0.001.


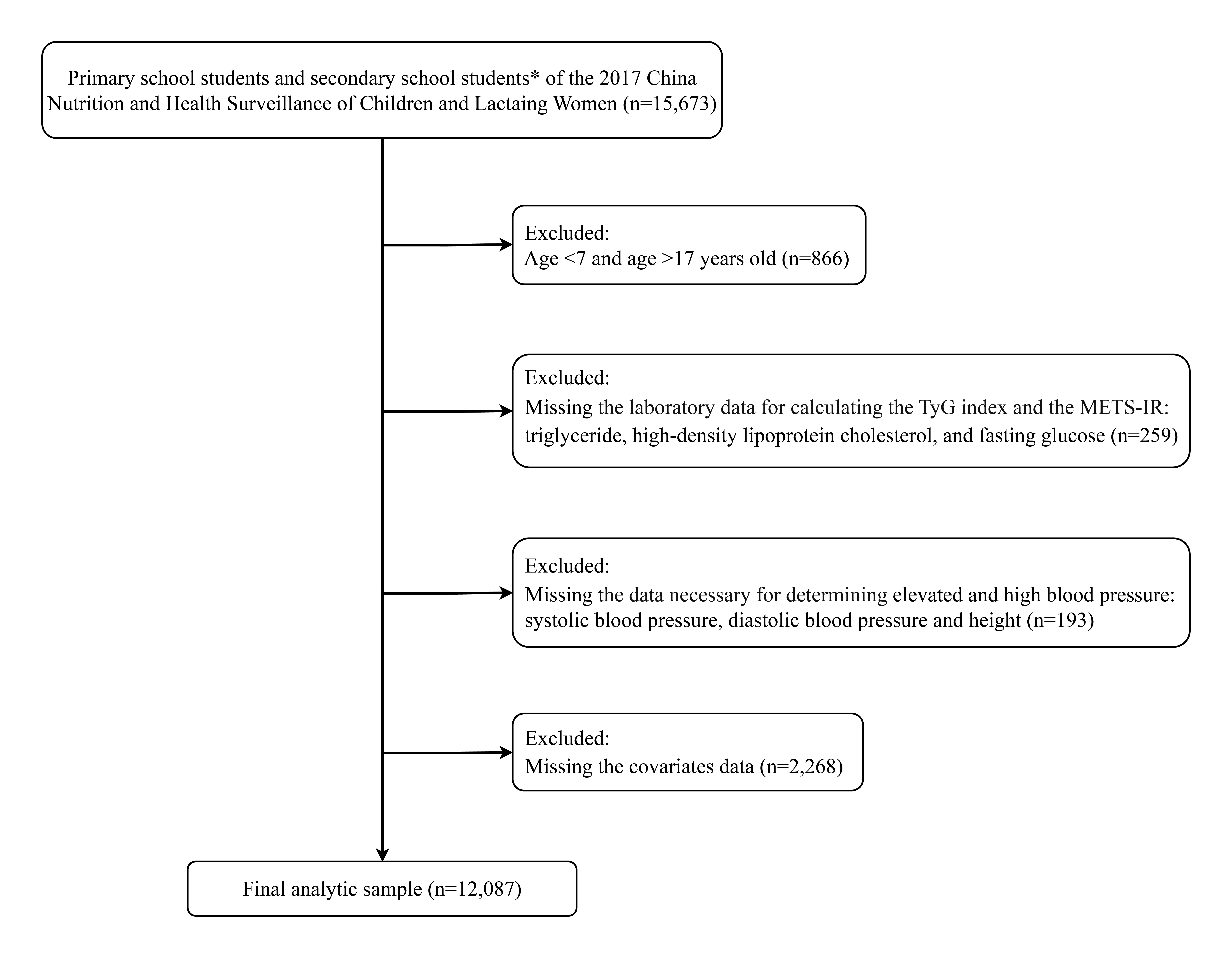


**Supplementary Figure 1.** Flowchart of inclusion and exclusion.

*Participants were from five provinces (Shandong, Jiangsu, Guangdong, Guizhou, Inner Mongolia) within the Surveillance coverage.


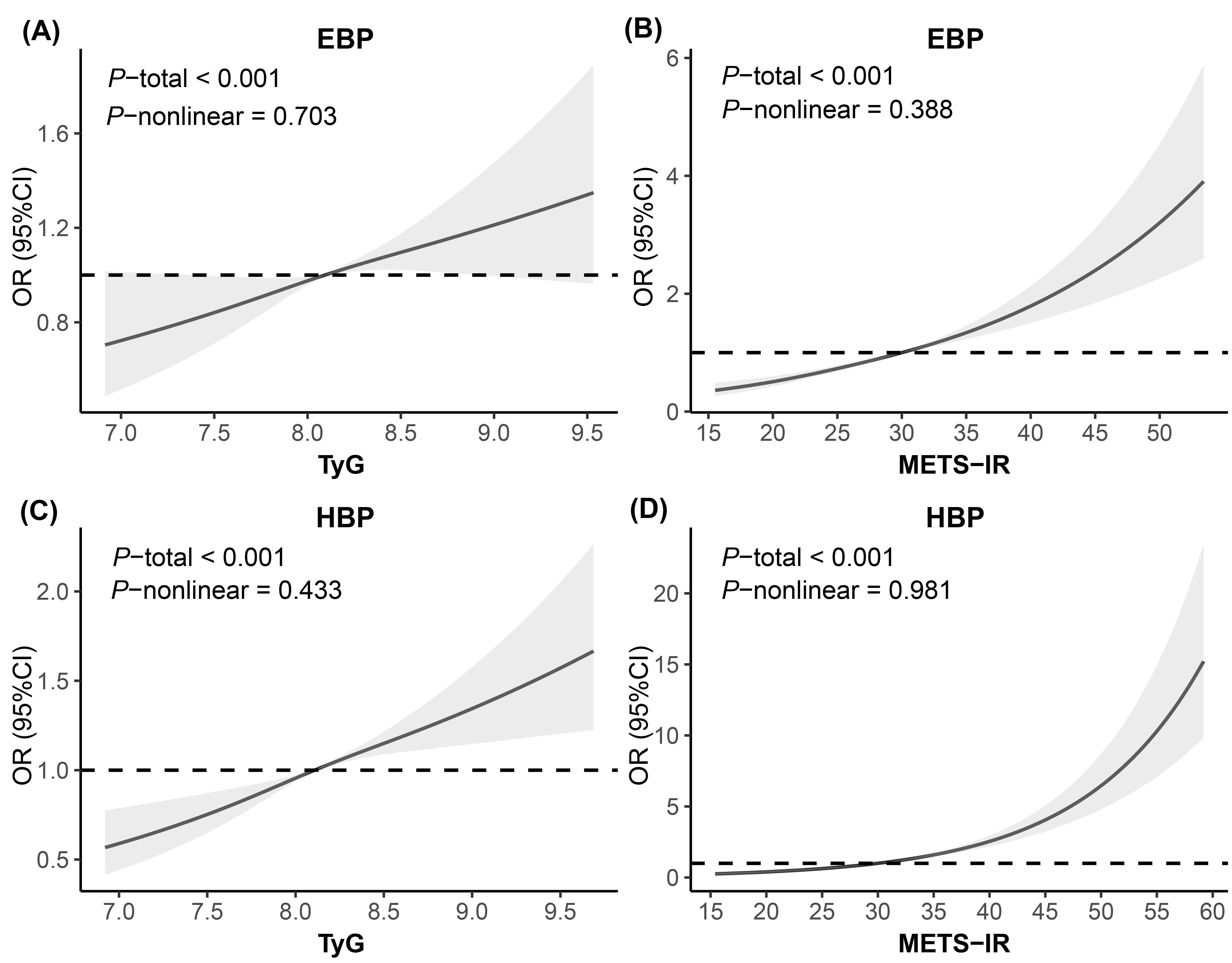


**Supplementary Figure 2.** Restricted cubic spline analysis for the relationship of insulin resistance indices with elevated blood pressure and high blood pressure. Knots were set to 3 for smooth curve fitting. Adjusted for age, sex, abdominal obesity, moderate-vigorous physical activity, sleep sufficiency, passive smoking, alcohol intake, parental education level, unhealthy dietary quality score, family history of hypertension, residence, serum uric acid, estimated glomerular filtration rate, total protein, total cholesterol, and low-density lipoprotein cholesterol. *OR*, odds ratio; *CI*, confidence interval; *EBP*, elevated blood pressure; *HBP*, high blood pressure; *TyG*, triglyceride-glucose index; *METS-IR*, metabolic score for insulin resistance.


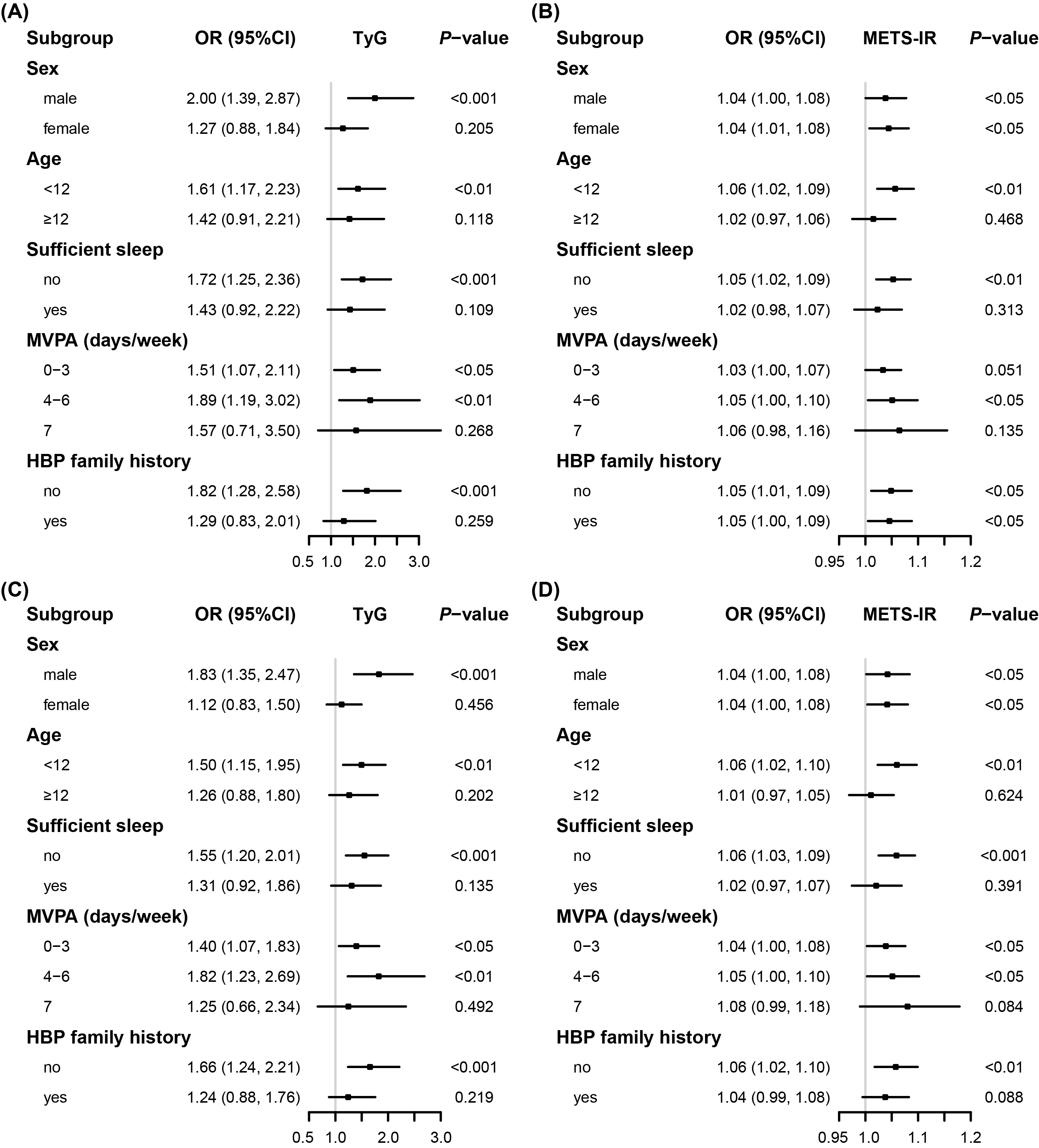


**Supplementary Figure 3.** Association between two insulin resistance indices and IDH stratified by different factors. (A) and (B) were adjusted for, if not stratified, age, sex, and abdominal obesity, using the entire dataset. (C) and (D) were adjusted for, if not stratified, age, sex, abdominal obesity, moderate-vigorous physical activity, sleep sufficiency, passive smoking, alcohol intake, parental education level, unhealthy dietary quality score, family history of hypertension, residence, serum uric acid, estimated glomerular filtration rate, total protein, and total cholesterol, using the dataset after propensity score matching. *OR*, odds ratio; *CI*, confidence interval; *HBP*, high blood pressure; *IDH*, isolated diastolic high blood pressure; *TyG*, triglyceride-glucose index; *METS-IR*, metabolic score for insulin resistance.
